# Glycosylation reactions mediated by hypervalent iodine: application to the synthesis of nucleosides and carbohydrates

**DOI:** 10.3762/bjoc.14.137

**Published:** 2018-06-28

**Authors:** Yuichi Yoshimura, Hideaki Wakamatsu, Yoshihiro Natori, Yukako Saito, Noriaki Minakawa

**Affiliations:** 1 Faculty of Pharmaceutical Sciences, Tohoku Medical and Pharmaceutical University, Komatsushima 4-4-1, Aoba-ku, Sendai, 981-8558, Japan; 2 Graduate School of Pharmaceutical Science, Tokushima University, Shomachi 1-78-1, Tokushima, 770-8505, Japan

**Keywords:** glycosylation, hypervalent iodine, Lewis acid, nucleoside, oligosaccharide

## Abstract

To synthesize nucleoside and oligosaccharide derivatives, we often use a glycosylation reaction to form a glycoside bond. Coupling reactions between a nucleobase and a sugar donor in the former case, and the reaction between an acceptor and a sugar donor of in the latter are carried out in the presence of an appropriate activator. As an activator of the glycosylation, a combination of a Lewis acid catalyst and a hypervalent iodine was developed for synthesizing 4’-thionucleosides, which could be applied for the synthesis of 4’-selenonucleosides as well. The extension of hypervalent iodine-mediated glycosylation allowed us to couple a nucleobase with cyclic allylsilanes and glycal derivatives to yield carbocyclic nucleosides and 2’,3’-unsaturated nucleosides, respectively. In addition, the combination of hypervalent iodine and Lewis acid could be used for the glycosylation of glycals and thioglycosides to produce disaccharides. In this paper, we review the use of hypervalent iodine-mediated glycosylation reactions for the synthesis of nucleosides and oligosaccharide derivatives.

## Introduction

Nucleic acids and oligosaccharides are both mandatory polymers for the maintenance of life and cell growth. The former exists in nuclei and codes genetic information, which is transformed into proteins through a transcription process known as the “central dogma” (i.e., DNA makes RNA makes proteins). The latter make up the cell walls of microorganisms and also play a role in transmitting information on the cell surface, whose interactions with proteins are a starting point for signal transduction into cells [1]. Since both types of polymers are essential for cell viability, their biological synthesis, including the synthesis of their monomer units, e.g., nucleotides, is highly regulated. Damage to these vital molecules often results in congenital disease with ultimately fatal consequences [2–3]. Accordingly, the study of polymers and their biosynthesis is quite important, and informs the development of new drugs for diseases including cancers and infectious diseases caused by viruses [4–7]. Indeed, many drugs related to nucleic acids and oligosaccharides have been developed and used in clinical fields. Synthetic chemists have contributed to the studies by supplying biological tools for the analyses of these polymers, as well as by synthesizing effective drug candidates for the diseases mentioned above [4,8–14].

To synthesize nucleoside and oligosaccharide derivatives, glycosylation reactions are often used to form a glycoside bond. In the case of nucleoside synthesis, a coupling reaction between a persilylated nucleobase and a sugar donor is typically used [15–17]. On the other hand, the reaction between an acceptor and sugar donor is carried out in the presence of an appropriate activator for oligosaccharide synthesis [18–19]. In both cases, a Lewis acid is generally used as an activator for sugar donors. Our previous review focused on the development of glycosylation reactions and their application to the synthesis of nucleoside derivatives [17]. In this review, we showed our glycosylation reactions under oxidative conditions. These were quite useful and the conceptually similar reactions were widely used for synthesizing nucleoside derivatives. Recently, a combination of a Lewis acid catalyst and hypervalent iodine was developed for synthesizing 4’-thionucleosides, which was based on a Pummerer-type reaction coupled with oxidation. The concept of the oxidative glycosylation reaction was successfully applied to the synthesis of other nucleoside derivatives, including 4’-selenonucleosides and carbocyclic nucleosides. The hypervalent iodine-mediated glycosylation has also been used for oligosaccharide synthesis employing glycals and thioglycosides as sugar donors. In this review, we survey the synthesis of nucleoside and disaccharide derivatives under oxidative conditions mostly based on the hypervalent iodine-mediated glycosylation reactions.

## Review

### Synthesis of 4’-thionucleosides

**Figure 1:**
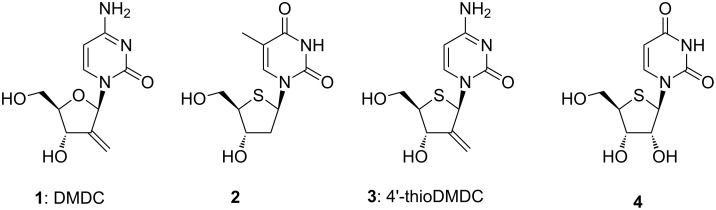
Design of potential antineoplastic nucleosides.

Over the last decade, we have steadily pursued the identification of novel antitumor and antiviral nucleoside derivatives [17,20–22]. Matsuda and co-workers reported a 2’-substituted cytidine derivative, DMDC (**1**), with potent antitumor activity [23–24]. In other reports, Walker [25] and Secrist [26] independently described the potent antiherpesvirus activity of 2’-deoxy-4’-thionucleoside **2**, in which sulfur was introduced in place of the sugar ring oxygen of 2’-deoxynucleoside. The results for 2’-substituted nucleosides and 2’-deoxy-4’-thionucleosides strongly suggested that 2’-substituted 4’-thionucleosides would be promising candidates for novel antitumor agents. Thus, we designed a novel 2’-substituted 4’-thiocytidine, 4’-thioDMDC (**3**), as our target molecule for potential antitumor agents [27–28] (Figure 1).

At the time we started our project, there had been no reports regarding the synthesis of even 2-substituted 4-thiosugar derivatives. We thus developed the first synthetic route accessing the 4-thiosugar derivative by way of bicyclic intermediate **8** from diacetoneglucose (**5**). Construction of the bicyclic ring of **8** was achieved by consecutive inter-/intramolecular S_N_2 reactions of the dimesylate derivative **7** obtained by manipulations of **5**. After acetal hydrolysis and the subsequent hydride reduction, 4-thioarabinose derivative **9** was obtained in good yield. Introduction of a TBDPS group at the primary hydroxy group of **9**, oxidation and Wittig reaction, followed by deprotection of the benzyl group, gave allyl alcohol **11**.

**Scheme 1:**
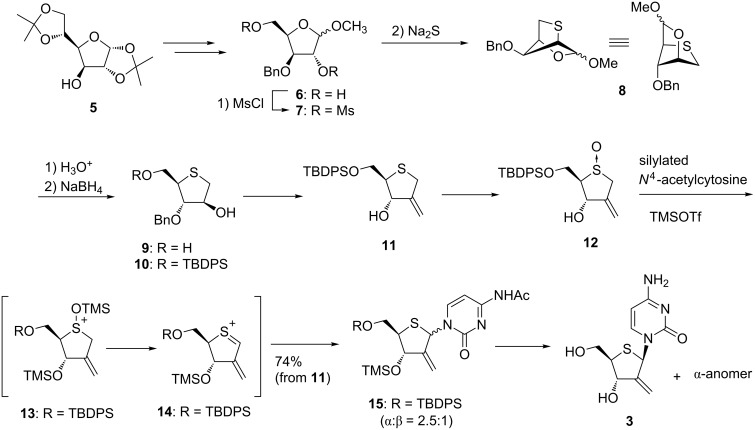
Synthesis of 4’-thioDMDC.

The most popular method to form a glycosyl bond between the sugar moiety and the base of a nucleoside is a Vorbrüggen reaction [15–16], in which a silylated base and sugar donor, e.g., 1-acetoxy sugar, are condensed by a Lewis acid catalyst. It was clear that this reaction could also be used in the synthesis of 4’-thionucleosides as well as normal “4’-oxy” nucleosides. However, for reasons which will be described later, we decided to develop an alternative method to build the glycosyl bond of 4’-thionucleosides by using a direct coupling of a 4-thiosugar sulfoxide and a silylated base under sila-Pummerer conditions [29–30]. We found that treatment of **12**, obtained by oxidation of **11**, with excess persilylated *N*^4^-acetylcytosine in the presence of TMSOTf as a Lewis acid gave an inseparable mixture of α- and β-anomers of 4’-thioDMDC derivatives **15** in good yield. Based on the study of the sila-Pummerer reaction by Kita, it was plausible that the reaction proceeded via the formation of sulfenium ion **14** which was formed by β-elimination of silylated sulfoxide **13**. The 4’-thioDMDC derivative **15** was deprotected and the resulting anomeric mixture was separated to furnish 4’-thioDMDC (**3**) and its α-anomer [27–28] (Scheme 1).

**Scheme 2:**
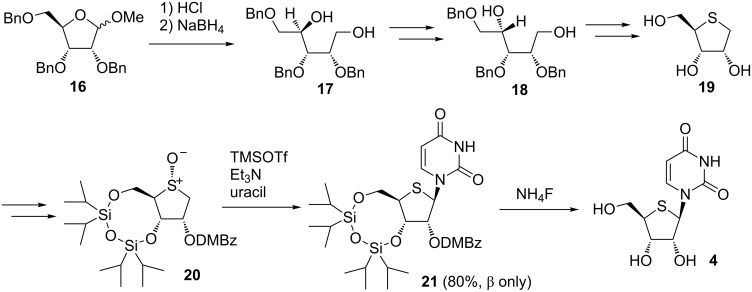
Synthesis of 4’-thioribonucleosides by Minakawa and Matsuda.

After we reported the synthesis of 4’-thioDMDC using a Pummerer-type glycosylation reaction, Minakawa and Matsuda applied the reaction to the syntheses of 4’-thioribonucleosides. Applying the synthetic scheme of 2’-deoxy-4’-thionucleoside by Walker to a ribo derivative, 2-dimethoxybenzoate **20** was prepared from tribenzylated ribose **16**. Introduction of a dimethoxybenzoyl (DMBz) group at the 2-position and diastereoselective formation of sulfoxide **20**, favored in Pummerer-type glycosylation reactions and cases where the approach of the nucleophile is restricted, were the key strategies for their synthesis of 4’-thioribonucleosides. Under optimized conditions, the desired 4’-thiouridine derivative **21** was the sole product and it was obtained in excellent yield (Scheme 2). Using the method developed, they succeeded in preparing all four kinds of 4’-thioribonucleosides [31].

**Scheme 3:**
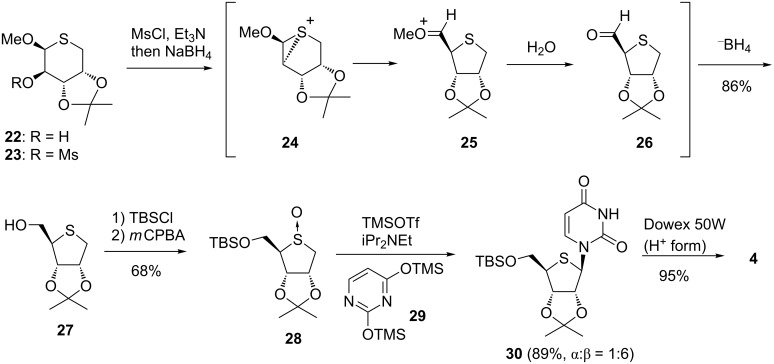
Synthesis of 4’-thioribonucleosides by Yoshimura.

We also synthesized 4’-thioribonucleosides constructing the skeleton of the 4-thioribose via a ring-contraction reaction under reductive conditions [32] from 2-mesylate **23**, which was obtained from **22**. As shown in Scheme 3, the reaction first started to form an episulfonium ion **24** triggered by intramolecular S_N_2 reaction at the 5-position by sulfur atom. Secondary, ring contraction from thiopyranose to thiofuranose occurred to produce 5-aldehyde **26**. Finally, hydride reduction of **26** gave the 4-thiofuranose derivative **27**. The Pummerer-type glycosylation reaction of 5-*O*-silylated sulfoxide **28**, by treating with 2,4-bis(trimethylsilyl)uracil (**29**) and excess diisopropylethylamine (DIPEA) in the presence of TMSOTf, gave 4’-thiouridine derivative **30** in a good yield. The reaction stereoselectively proceeded and resulted the predominant formation of the β-anomer due to steric hindrance of the 2,3-di-*O*-isopropylidene group.

**Figure 2:**
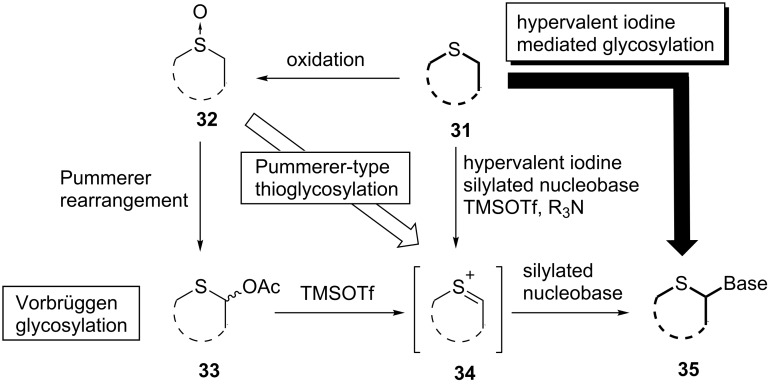
Concept of the Pummerer-type glycosylation and hypervalent iodine-mediated glycosylation.

Before our reports regarding the Pummerer-type glycosylation, the synthesis of 4’-thionucleosides was based on the known chemistry: typically, a 1-acetoxy-4-thiosugar or its synthetic equivalent was obtained from natural sugars and subjected to the Vorbrüggen reaction as in the case of 2’-deoxy-4’-thionucleosides [25–26]. When synthesizing 4’-thionucleosides by the way of a sulfide derivative **31**, the known chemistry should lead us to use a classical Pummerer reaction to produce 1-acetoxy derivative **33** after converting **31** to the corresponding sulfoxide **32**. Even though this scheme should be promising enough, we intended to introduce an additional synthetic idea based on the fact that both of the reaction intermediate of the Vorbrüggen reaction [15–16] of **33** and the sila-Pummerer reaction developed by Kita [29–30] involving sulfoxide **32** would be the same sulfenium ion **34**. This new glycosylation reaction was unique and attractive since it was capable of skipping a step. In other words, the reaction could directly access sulfenium ion **34** from sulfoxide **32**. Thus we developed the Pummerer-type glycosylation as mentioned above. From these results it can be deduced that the expected sulfenium ion had formed and that the concept of the Pummerer-type glycosylation was actually effective for the formation of the glycosyl bond of 4’-thionucleosides. After we had reported our synthesis of 4’-thioDMDC, the method was widely adopted for the synthesis of 4’-thionucleoside derivatives by other groups and became a standard approach for the glycosylation [33–37]. On the other hand, the conversion from the sulfide to 4’-thionucleoside using the Pummerer-type glycosylation included an oxidation step. If the oxidation of sulfide **31** and the Pummerer-type glycosylation of the sulfoxide **32** could be performed in the same flask, the reaction could bypass two of the reaction steps and would directly produce 4’-thionucleoside **35** from **31**. Indeed, the utilization of hypervalent iodine would have enabled this short-cut reaction (Figure 2).

**Scheme 4:**
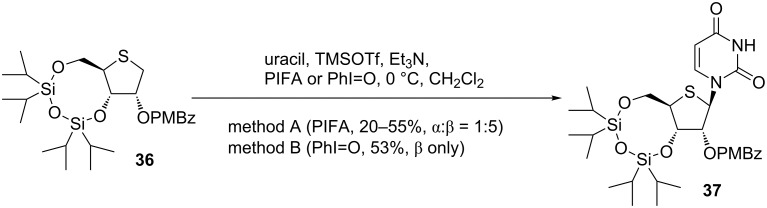
Oxidative glycosylation of 4-thioribose mediated by hypervalent iodine.

Hypervalent iodine reagents have been widely used in organic synthesis [38]. Although originally used as oxidative agents, their use has spread to coupling reactions, including those for the formation of C–C bonds [39–43]. In the case of C–N bond formation, introduction of an azido group using PhI=O and TMSN_3_ was reported by Kita and co-workers [44]. Their paper prompted Nishizono et al. to study the glycosylation reaction for 4’-thionucleosides using hypervalent iodine reagents. As a 4-thiosugar donor, 2-*p*-methoxybenzoate derivative **36** was prepared following Matsuda’s method as shown in Scheme 2, and then was subjected to the Pummerer-type glycosylation mediated by hypervalent iodine. Treatment of **36** with bis(trifluoroacetoxy)iodobenzene (PIFA) and uracil in the presence of trimethylsilyl trifluoromethanesulfonate (TMSOTf) and triethylamine gave a 5:1 mixture of 4’-thiouridine derivative **37** in 55% yield. The reaction of **36** with iodosylbenzene (PhI=O) proceeded stereoselectively and gave only the β-anomer of **37** in 53% yield [45] (Scheme 4).

**Figure 3:**
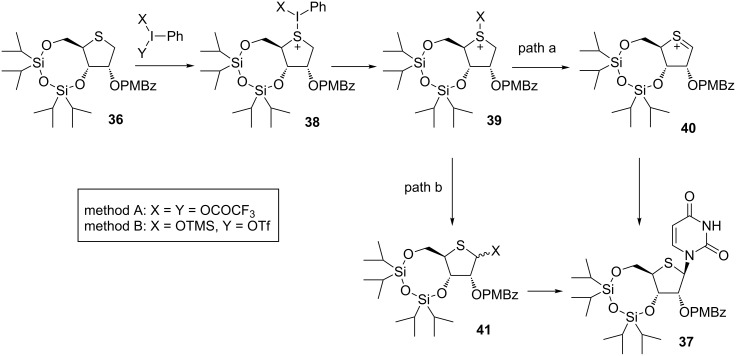
Speculated mechanism of oxidative glycosylation mediated by hypervalent iodine.

The mechanism of hypervalent iodine-mediated glycosylation can be expressed as shown in Figure 3. The activated hypervalent iodine reagents in the presence of TMSOTf reacted a sulfur atom of **36** to give **38**, in which elimination of iodobenzene and HX might subsequently occur to generate a sulfenium ion **40** (path a). The nucleophilic attack of the silylated base to the sulfenium ion **40** favored approaching from the β-face to give only the β-anomer **37** as in the case of Minakawa and Matsuda’s synthesis described above.

Nishizono considered that the difference between the stereoselectivities of the coupling reactions in methods A and B was caused by the existence of another reaction path of the sulfonium salt (**38** or **39**). In path b, the 4-thiosugar **41** was generated and reacted with a nucleobase, giving a mixture of α- and β-anomers since the reaction might occur by the simple S_N_2 reaction. Thus, the reaction proceeded through both paths a and b in method A, but path a was predominant in the reaction of method B [45] (Figure 3).

**Scheme 5:**
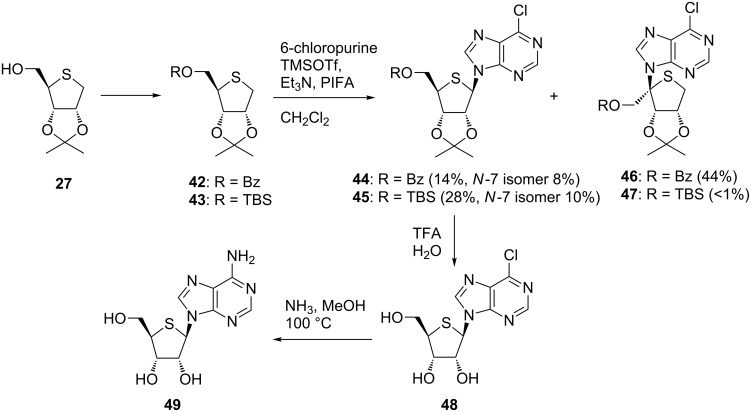
Synthesis of purine 4’-thioribonucleosides using hypervalent iodine-mediated glycosylation.

Nishizono et al. applied the hypervalent iodine-mediated glycosylation to purine 4’-thionucleosides [46]. However, the reaction of **36** with 6-chloropurine resulted in the formation of a regioisomer reacting at the 4-position without any formation of the desired purine 4’-thionucleoside. The result should relate to the acidity of the α hydrogen adjacent to a sulfur atom, which affects the regioselectivity of the reaction. To study the effects of a protecting group on the reaction, the regioselectivity of the reaction was examined using **42** and **43**, which were obtained from **27**. When the 5-hydroxy group was protected with a benzoyl group, the coupling reaction of **42** occurred at the 4-position, as in the case mentioned above, to give **46** in 44% yield along with the desired product and its N7 isomer. In contrast, switching the protecting group of **27** at the 5-position to TBS resulted in the exclusive formation of **45** reacted at the 1-position (28%) along with the N7 stereoisomer (10%). These results support the above-mentioned hypothesis. Finally, 4'-thioadenosine (**49**) was synthesized by treating **45** with TFA followed by methanolic ammonia [46] (Scheme 5).

**Scheme 6:**
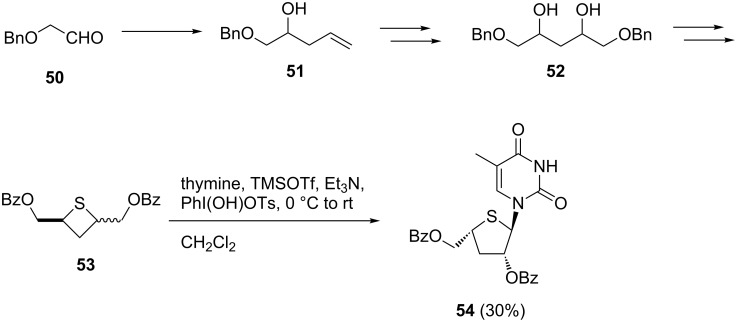
Unexpected glycosylation of a thietanose derivative.

The same group attempted to apply the oxidative coupling reaction to the synthesis of thietane nucleosides [47]. The substrate of the coupling reaction was prepared as shown in Scheme 6 starting from benzyloxyacetaldehyde (**50**). When a hypervalent iodine reagent was used for glycosylation with a diastereomeric mixture of sulfide **53**, the reaction stereoselectively gave the ring-expanded nucleoside **54** in 30% yield, but did not give the desired thietane nucleoside at all (Scheme 6).

**Scheme 7:**
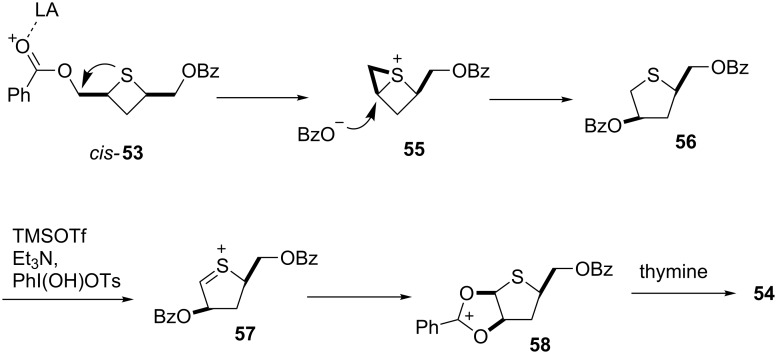
Speculated mechanism of the ring expansion of a thietanose derivative.

Considered that the ring-expansion occurred in the absence of the hypervalent iodine reagent, the Nishizono and co-workers speculated that the reaction mechanism was as shown in Scheme 7. First, the Lewis acid catalyzed the intramolecular S_N_2 reaction of sulfur to form the epi-sulfonium ion **55**, which proceeded only from the *cis*-isomer due to the steric requirement. The subsequent nucleophilic attack leaving the benzoate anion resulted in the formation of a ring-expanded product **56**, which became a substrate of the hypervalent iodine-mediated glycosylation. As a result, 4’-thiofurano nucleoside **54** was stereoselectively obtained with the assistance of the neighboring benzoyl group as in **58**.

**Scheme 8:**
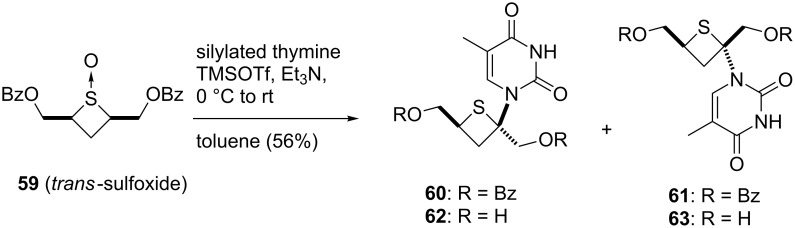
Synthesis of thietanonucleosides using the Pummerer-type glycosylation.

The desired thietanonucleosides **62** and **63** with an anomeric hydroxymethyl group were synthesized by the Pummerer-type glycosylation reaction of *trans-*cyclobutane sulfoxide **59**. The authors concluded that the stereochemistry of the sulfoxide and the nature of the protecting groups had no significant effect on the yield of the Pummerer-type glycosylation [47] (Scheme 8).

Pummerer-type glycosylation, which was developed by our group, improved the synthesis of 4’-thionucleosides. It greatly contributed to search new biological active nucleoside derivatives. The use of hypervalent iodine reagents helped to further improve their synthesis by saving reaction steps to improve synthetic efficiency.

### Synthesis of 4’-selenonucleosides

The unique biological activity of 4’-thionucleosides triggered the synthesis of their chalcogen isosters, 4’-selenonucleosides, the activity of which were reported. The first synthesis of 4’-selenonucleosides was reported by Jeong and co-workers in 2008 [48–49].

**Scheme 9:**
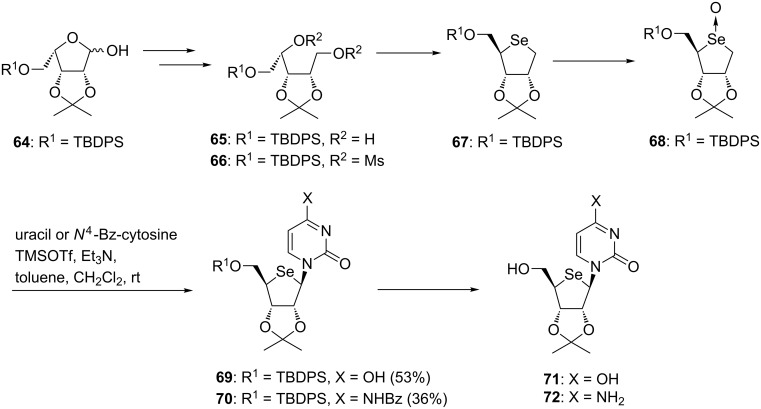
First synthesis of 4’-selenonucleosides.

As in the case of the 4’-thioribonucleoside described in Scheme 3, Jeong et al. chose a 2,3-di-*O*-isopropylidene-protected intermediate as a donor of glycosylation, which was synthesized based on their method developed for 4’-thionucleosides. Starting from compound **64**, which was obtained from D-gulonic γ-lactone, dimesylate **66** was prepared. The consecutive inter-/intramolecular S_N_2 reactions of **66** by selenide anion gave a 4-seleno sugar **67** in an excellent yield. After converting **67** to the corresponding selenoxide, the resulting **68** was immediately treated with uracil or *N*^4^-benzoylcytosine under the same conditions for Pummerer-type glycosylation to give the desired 4’-selenouridine and 4’-selenocytidine derivatives in moderate yields. Deprotection of the nucleoside derivatives afforded 4’-selenouridine and 4’-selenocytidine, respectively [48] (Scheme 9). In the year in which the first synthesis of 4’-selenonucleoside was reported, Jayakanthan et al. used the same strategy to synthesize 4’-selenonucleosides, including 4’-selenoadenosine [50].

**Scheme 10:**
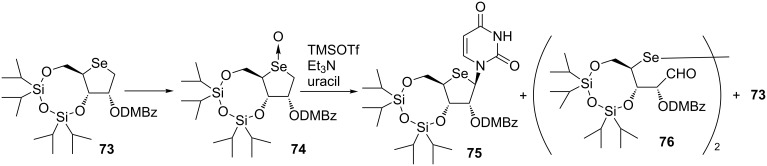
The Pummerer-type glycosylation of 4-selenoxide **74**.

After successful application of the Pummerer-type glycosylation to the synthesis of 4’-selenonucleosides, Jeong’s group reported various 4’-selenonucleoside derivatives by using the same method [51–58]. Minakawa and his group attempted to synthesize 4’-selenonucleosides based on their method described in Scheme 2 [59]. However, the Pummerer-type glycosylation of selenoxide **74** obtained from **73** gave the desired 4’-selenonucleoside in low yield along with the formation of diselenide **76** and deoxygenated **73** (Scheme 10). One of the reasons for the unsatisfactory result was the instability of selenoxide **74**. Jeong et al. faced the same problem and suppressed decomposition by the immediate reaction after synthesizing the corresponding selenoxide [48].

To overcome these problems, Minakawa decided to use hypervalent iodine for the glycosylation reaction [59] as in Nishizono’s synthesis of 4’-thionucleosides [45]. First, they optimized the reaction conditions by examining the reaction of **73** with uracil in the presence of hypervalent iodine reagents. None of the desired pyrimidine nucleoside **75** was formed when the reaction was performed by treatment with iodosylbenzene, TMSOTf and triethylamine in the presence of the silylated uracil (Table 1, entry 1). Instead of trimethylamine, 2,6-lutidine was employed to give **75** in 48% yield together with selenoxide **74** (20%) and starting **73** (8%) (Table 1, entry 2). The use of more reactive hypervalent iodine agents (PIFA and diacetoxyiodobenzene) did not improve the chemical yield of **75** (Table 1, entries 3 and 4). When **73** was treated with iodosylbenzene, TMSOTf, 2,6-lutidine and the silylated uracil in dichloroethane at 50 °C, the reaction gave **75** in 64% yield while suppressing the formation of **74** (Table 1, entry 5).

**Table 1:** The Pummerer-like glycosylation reaction mediated by hypervalent iodine.

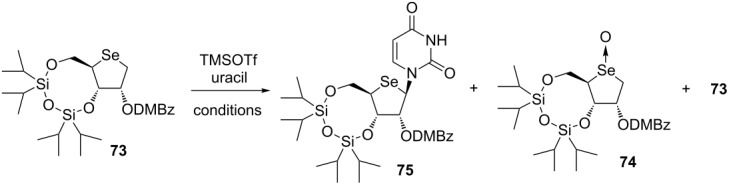								

	Conditions					Yield (%)		

Entry	Hypervalent iodine (1.2 equiv)	Base (8 equiv)	Solvent	Temp (°C)	Time (h)	**75**	**74**	**73**

1	PhIO	Et_3_N	CH_2_Cl_2_	0	4.5	0	0	33
2	PhIO	2,6-lutidine	CH_2_Cl_2_	rt	17	48	20	8
3	PhI(OCOCF_3_)_2_	2,6-lutidine	CH_2_Cl_2_	rt	3	38	0	40
4	PhI(OAc)_2_	2,6-lutidine	CH_2_Cl_2_	rt	5	25	0	20
5	PhIO	2,6-lutidine	ClCH_2_CH_2_Cl	50	1.5	64	0	13

Minakawa’s group attempted to apply the aforementioned reaction to the synthesis of purine derivatives [60]. Based on the reports by Jeong et al., who synthesized 4’-selenoadenosine using the Vorbrüggen reaction [53], they conceived that the hypervalent iodine-mediated reaction of “disarmed” sugar donor **73** bearing an electron-withdrawing group at the 2-position would not readily yield the desired purine derivative. Therefore, they decided to use “armed” seleno sugar **67** as a donor for the hypervalent iodine-mediated glycosylation reaction as in Jeong’s synthesis.

**Scheme 11:**
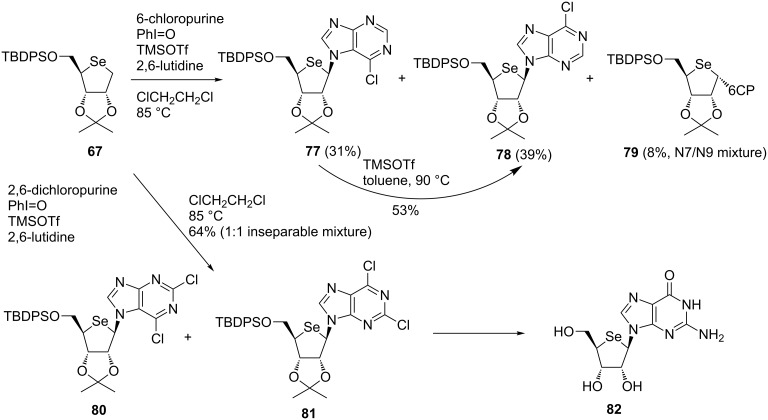
Synthesis of purine 4’-selenonucleosides using hypervalent iodine-mediated glycosylation.

The reaction of **67** was performed by treating with silylated 6-chloropurine, iodosylbenzene, TMSOTf and 2,6-lutidine in dichloroethane at 85 °C for 2.5 h to give the desired N9-isomer **78** in 39% yield along with the formation of the N7-isomer **77** (31%) and the α-isomer (8%, N7/N9 mixture). On the other hand, consumption of **67** required longer times and subsequent isomerization to **78** was insufficient at 50 °C, giving **78** in 31% yield with the predominant formation of **77** (40%). The separated N7 isomer **77** was successfully isomerized to the desired N9 isomer **78** in 53% yield upon treatment with TMSOTf in toluene at 90 °C. Under similar conditions, the hypervalent iodine-mediated glycosylation reaction of **67** in the presence of 2,6-dichloropurine was conducted. The coupling reaction proceeded to give an inseparable mixture of N7-isomer **80** and N9-isomer **81** in 64% yield (**80**:**81** = 1:1). To isomerize the undesired N7-isomer to the desired product as in the case of 2,6-dichloropurine, the subsequent treatment of the resulting mixture with TMSOTf in toluene at 90 °C gave rise to exclusive formation of the desired N9-isomer **81** in 62% yield. Finally, **81** was converted to the desired guanosine derivative **82** [60] (Scheme 11).

As in the case of 4’-thionucleosides, the use of hypervalent iodine greatly improved the glycosylation reaction with 4-seleosugars by skipping the preparation of unstable selenoxide derivatives.

### Synthesis of carbocyclic nucleosides

**Figure 4:**
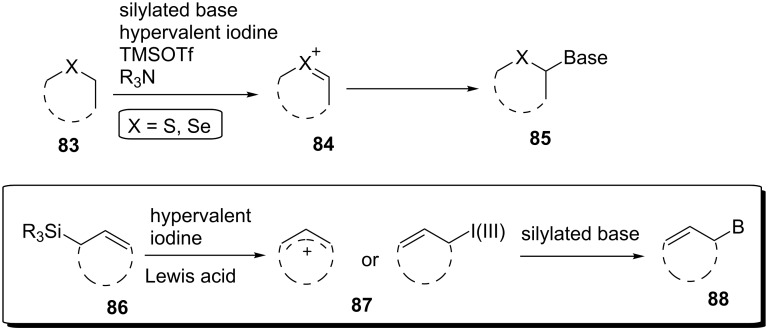
Concept of the oxidative coupling reaction applicable to the synthesis of carbocyclic nucleosides.

As described above, in the hypervalent iodine-mediated glycosylation, a thiosugar donor **83** was oxidized to a cationic intermediate **84** with the assistance of a Lewis acid (TMSOTf) and a base and the subsequent nucleophilic attack of silylated base to **84** gave the desired nucleoside **85**. The success of the hypervalent iodine-mediated glycosylation led us to apply the reaction to the synthesis of carbocyclic nucleosides. In addition, we were also encouraged by the study of Ochiai, who developed the Friedel–Crafts reaction via umpolung of allylsilanes using hypervalent-iodine reagents [61] and the pioneering work on C–N bond formation using hypervalent iodine by Kita [62]. Thus, we envisioned the use of allylsilanes as a pseudosugar donor for the synthesis of carbocyclic nucleosides. We expected to couple a cyclic allylsilane **86**, which could act as a pseudosugar donor for carbocyclic nucleosides **88**, with a persilylated nucleobase by using a combination of hypervalent iodine and an appropriate Lewis acid (Figure 4).

**Scheme 12:**
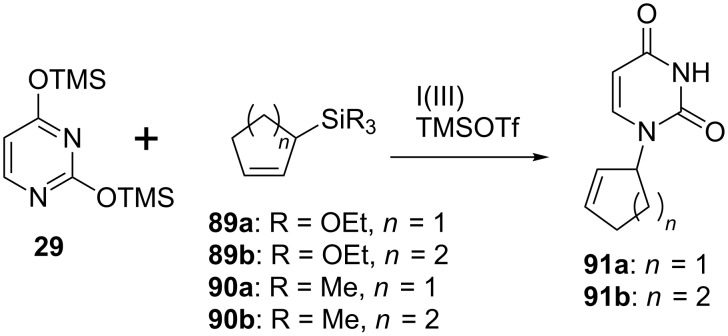
Oxidative coupling reaction mediated by hypervalent iodine.

As shown in Scheme 12, an oxidative coupling reaction was examined using a model reaction [63]. Cycloalkenylsilanes **89a**,**b** and **90a**,**b** were prepared by hydrosilylation of cyclopentadiene and cyclohexadiene. Using TMSOTf as a Lewis acid, the hypervalent iodine-mediated coupling reaction of **89a**,**b** and **90a**,**b** with silylated uracil **29** was examined and the results are summarized in Table 2. Our first attempt to couple triethoxysilanes **89a**,**b** with **29** in the presence of diacetoxyiodobenzene gave cycloalkenyluracil **91a** and **91b** in 45% and 49% yields respectively (Table 2, entries 1 and 2). On the other hand, the use of trialkylsilanes **90a** and **90b** successfully improved the chemical yield of **91a** and **91b** (Table 2, entries 3 and 4). In contrast, the reactions using PIFA, iodosylbenzene, and [hydroxyl(tosyloxy)iodo]benzene (PhI(OH)OTs) resulted in a decrease of the reaction yield (Table 2, entries 5–7).

**Table 2:** Summary of the oxidative coupling reaction using hypervalent iodine.

entry	comp	I(III)	Time (h)	yield (%)

1	**89a**	PhI(OAc)_2_	15	**91a**: 45
2	**89b**	PhI(OAc)_2_	15	**91b**: 49
3	**90a**	PhI(OAc)_2_	1	**91a**: 65
4	**90b**	PhI(OAc)_2_	1	**91b**: 65
5	**90b**	PhI(O_2_CCF_3_)_2_	1	**91b**: 55
6	**90b**	PhIO	1	**91b**: 57
7	**90b**	PhI(OH)OTs	1	**91b**: 29

To prove the usefulness of the oxidative coupling reaction mediated by hypervalent iodine, the reaction was applied to the synthesis of a carbocyclic nucleoside derivative designed as a potential anti-HIV agent.

**Scheme 13:**
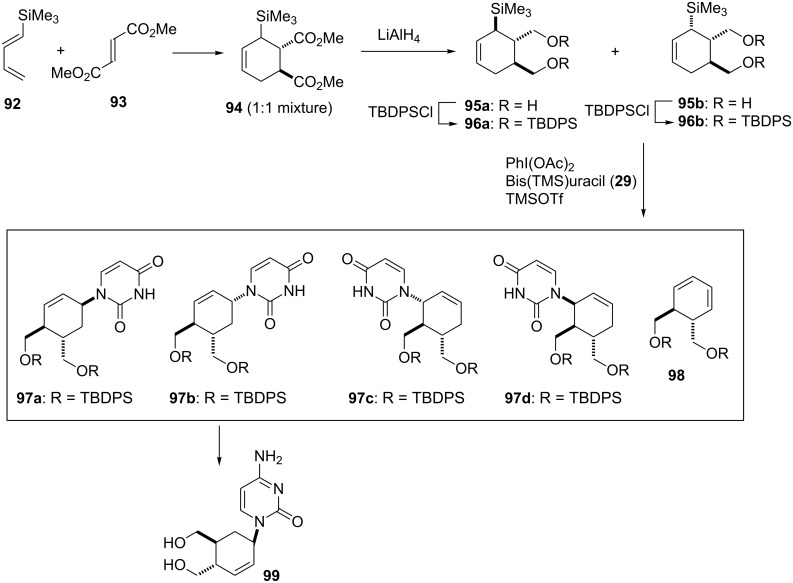
Synthesis of cyclohexenyl nucleosides using an oxidative coupling reaction.

As a target, cyclohexenylcytosine **99** was designed and was planned to synthesize using the oxidative coupling reaction. To prepare the substrate of the coupling reaction, cyclohexenylsilane **96** was synthesized using the Diels–Alder reaction of trimethylsilylbutadiene **92** and dimethyl fumarate (**93**), which gave cyclohexene diester **94** (1:1 mixture of diastereomers) [64]. Reduction and subsequent separation by silica gel column chromatography gave diols **95a** and **95b**, the hydroxy groups of which were protected to give di-TBDPS derivatives **96a** and **96b**. The resulting cyclohexenylsilanes **96a** and **96b** were subjected to the oxidative coupling reaction with 2,4-bis(trimethylsilyl)uracil (**29**) using diacetoxyiodobenzene, respectively, and the results are shown in Table 3. The reaction of **96a** gave an inseparable mixture containing 4 stereoisomers of **97a–d** with a ratio of 6:10:2:1.5, which was determined based on the analysis of its ^1^H NMR spectrum. The reaction of **96b** also gave a similar result. In both reactions, the formation of cyclohexadiene **98** was observed. These results strongly supported that the reaction proceeded through the carbocation intermediate, as expected and depicted in Figure 4, since **98** was considered to be formed by E1 elimination of the allyl cation intermediate. The fact that **96a** and **96b** showed different reactivities could be explained by the steric interaction between the substituents on the cyclohexene ring and the nucleobase approaching. Compounds **97a–d** were converted to the corresponding cytosine analogues [63]. During the course of conversion, all the stereoisomers were separated. Among them, only the cytosine derivative **99** showed weak anti-HIV activity (Scheme 13 and Table 3).

**Table 3:** Summary of the oxidative coupling reactions of **96a** and **96b**.

comp	time	yield (%)			ratio

		**97a–d**	**98**	recov.	**97a**:**97b**:**97c**:**97d**

**96a**	1 h	60	18	0	6:10:2.0:1.5
**96b**	24 h	50	11	20	3:10:2.5:0.5

An oxidative coupling reaction for synthesizing carbocyclic nucleosides mediated by hypervalent iodine was developed. Since the Friedel–Crafts type reaction involved carbocation intermediate, the reaction always gave a mixture of products. Unfortunately, the reaction was not efficient. However, it is worthy that the oxidative coupling reaction contains a novel type of C–N bond formation and would help to synthesize new carbocyclic nucleosides.

### Synthesis of dihydropyranonucleosides

**Figure 5:**
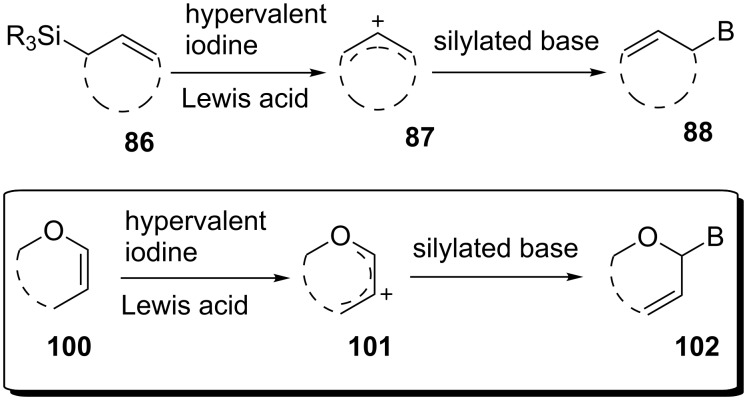
Concept of the oxidative coupling reaction of glycal derivatives.

The success of the oxidative coupling reaction for constructing a carbocyclic nucleoside skeleton led us to develop a glycosylation reaction applicable to glycal derivatives. Since an electron-rich enol ether unit of glycal could react with oxidative agents, it was expected to form a cationic intermediate as in the case of allylsilanes described above. A direct coupling of glycals with nucleobases is challenging, since it is formally a C–N bond-forming reaction with cleaving of the inactive C–H bond at the γ-position. Actually, the C–N bond-forming reactions using hypervalent iodine agents have attracted much attention [62,65–68]. In the case of the hypervalent iodine-catalyzed coupling reaction with allylsilanes (Figure 5), the reaction involves the following 2 steps: 1) the generation of allyl cation **87** by the oxidation of an allylsilane **86** with PhI(OAc)_2_ and TMSOTf, and 2) the subsequent nucleophilic attack of the persilylated base to **87** as shown in Figure 5. Therefore, we expected that subjecting the electron-rich glycal **100** to the hypervalent iodine-mediated reaction described above would generate an oxocarbenium ion **101** to serve as an intermediate, giving a nucleoside **102**.

**Scheme 14:**
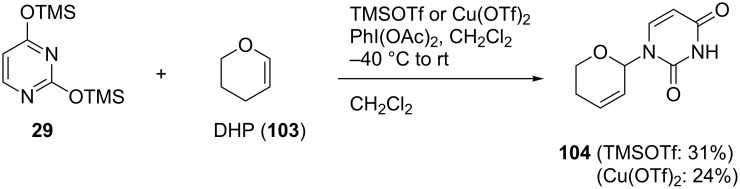
Oxidative coupling reaction of silylated uracil and DHP using hypervalent iodine.

First, we attempted model reactions of the oxidative coupling to enol ether using a TMSOTf/PhI(OAc)_2_ system. After several attempts, we found that the reaction of 3,4-dihydro-2*H*-pyran (DHP, **103**) with PhI(OAc)_2_ and TMSOTf, starting at −40 °C and then gradually raised to room temperature, gave a dihydropyranyluracil derivative **104** in 31% yield [69]. We also found that when Cu(OTf)_2_ was used as a catalyst in place of TMSOTf, the reaction gave **104** in 24% yield (Scheme 14).

**Scheme 15:**
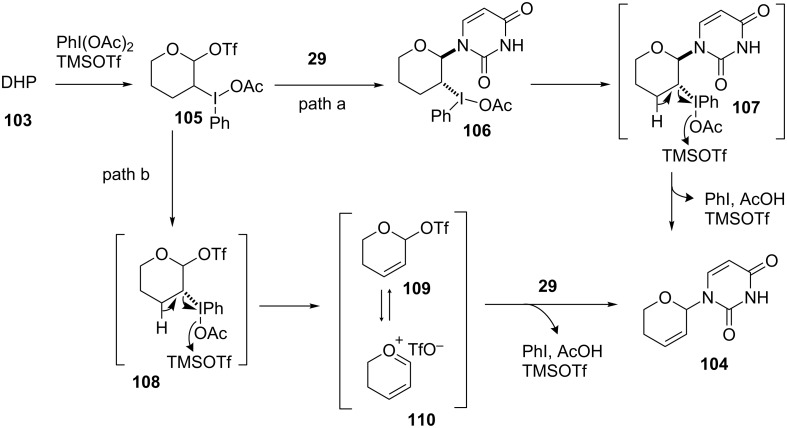
Proposed mechanism of the oxidative coupling reaction mediated by hypervalent iodine.

We speculated that the mechanism of the oxidative coupling reaction was as shown in Scheme 15. DHP (**103**) was reacted with PhI(OAc)_2_ to produce an acetoxyiodobenzene derivative **105** with the assistance of TMSOTf. With respect to the pathway from the intermediate **105** to the N1-substituted uracil **104**, there were two plausible routes. In path a, a nucleophilic attack of 2,4-bis(trimethylsilyl)uracil (**29**) occurs prior to an elimination. In path b, on the other hand, an allylic carbocation **110** formed from **108** reacts with **29**. From the result that the reaction of 2,3-dihydrofuran gave side products generated from an intermediate resembling **107** (data not shown), it was strongly suggested that the oxidative coupling reaction preferred path a rather than path b (Scheme 15).

**Figure 6:**
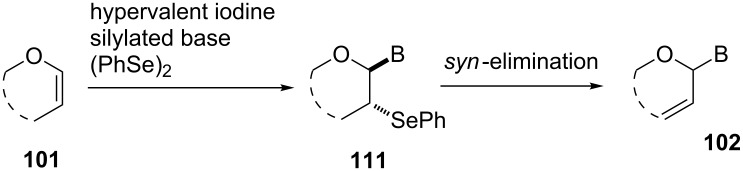
Synthesis of 2’,3’-unsaturated nucleosides using hypervalent iodine and a co-catalyst.

Because further optimization of the oxidative coupling reaction was not successful, we decided to examine the effect of adding a co-catalyst. The speculated reaction mechanism depicted in Scheme 15 suggested that the instability of the intermediates **105** and **106** might have caused the low yield of the oxidative coupling. Based on this idea, we intended to use (PhSe)_2_ as a co-catalyst, since it might prevent the formation of unstable **105** and **106** and yield **102** in one step (Figure 6).

We examined the effect of (PhSe)_2_ as an additive by the reaction of various glycals and their chemical equivalents [69] and the results are summarized in Table 4. The reaction of **103** and **29** was performed by treatment with PhI(OAc)_2_ and (PhSe)_2_ in the presence of catalytic amounts of TMSOTf to selectively yield a *trans*-isomer of 1-(3-phenylselanyltetrahydropyran-2-yl)uracil (**116**) in 73% yield (Table 4, entry 1). Although this result was unexpected, it was important, since the reaction appeared to be applicable to access various nucleoside derivatives, including 2’-deoxynucleosides. More importantly, we could avoid the use of unstable reagents such as PhSeBr. In other words, the reaction using hypervalent iodine and stable (PhSe)_2_ in the presence of a Lewis acid would be expected to yield the same products as the reaction using PhSeBr. The reaction with dihydrofuran (**112**) furnished 1-(3-phenylselanyltetrahydrofuran-2-yl)uracil (**117**) in 31% yield (Table 4, entry 2). The reaction of **113** with **29** at −5 °C afforded **118** in 69% yield (Table 4, entry 3). The reaction of **114** gave an anomeric mixture of **119** in 80% yield with the predominant formation of the β-nucleoside (Table 4, entry 4). In contrast, the oxidative glycosylation reaction of D-glucal **115** gave a 1:1 mixture of α-**120** and β-**120** in 64% yield (Table 4, entry 5). From these data, the oxidative coupling reaction mediated by hypervalent iodine of glycal derivatives can clearly be regarded as a new glycosylation reaction that is applicable to the synthesis of 2’-deoxy- and 2’,3’-dideoxydidehydronucleosides, some of which are known to have anti-HIV activity (Table 4).

**Table 4:** Summary of the oxidative coupling reaction of bis(trimethylsilyl)uracil **29** with enol ethers using the TMSOTf/PhI(OAc)_2_/(PhSe)_2_ system.

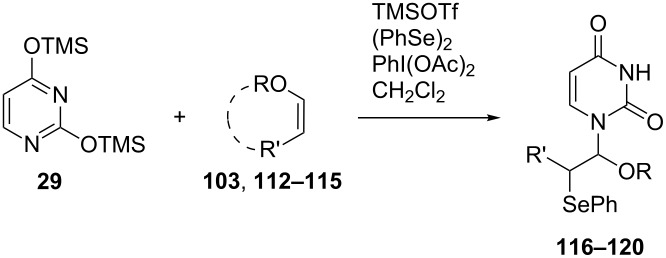			

entry	enol ether	product	yield (%)

1	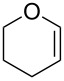 **103**	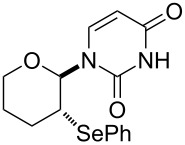 **116**	73
2	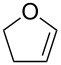 **112**	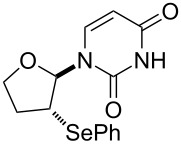 **117**	31
3	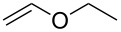 **113**	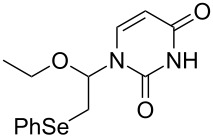 **118**	69
4	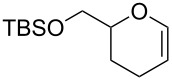 **114**	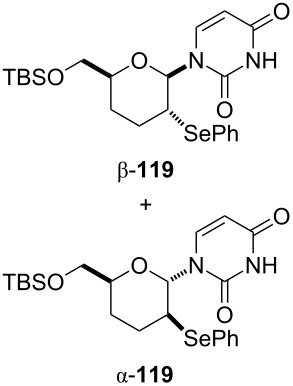	80 (α:β=1:2)
5	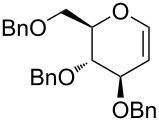 **115**	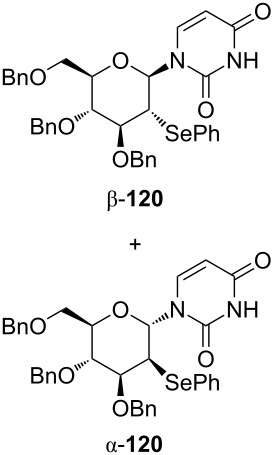	64 (α:β=1:1)

**Scheme 16:**
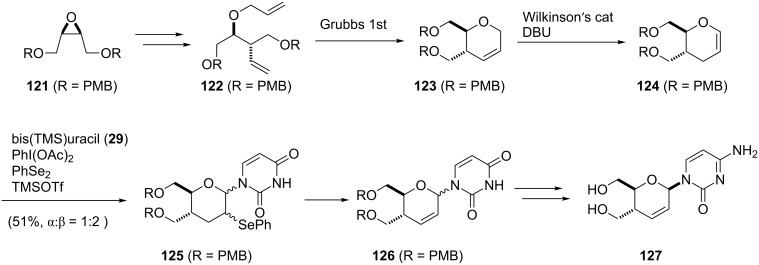
Synthesis of dihydropyranonucleoside.

To reveal the scope of this reaction, we designed a new dihydropyranonucleoside as a potential anti-HIV agent and attempted to synthesize it by using the oxidative coupling reaction [70]. First, the PMB-protected epoxide **121** was converted to diene **122**. The dihydropyran ring of **123** was constructed by RCM of **122** catalyzed by a Grubbs 1st generation catalyst. The isomerization of the double bond in **123** by treatment with a Wilkinson catalyst under basic conditions afforded glycal **124** (Scheme 16).

The hypervalent iodine-mediated glycosylation of 2,4-bis(trimethylsilyl)uracil (**29**) with glycal **124** gave an inseparable mixture of α- and β-anomers **125** (α:β = 1:2) in 51% yield as we expected. Compound **125** was then oxidized by treatment with mCPBA, followed by elimination of the resulting selenoxide to give **126**. After the separation of anomers, the major β-anomer was converted into a cytosine derivative **127** [70]. However, **127** did not show any activity against HIV whereas its 5’-thio counterpart did show anti-HIV activity (Scheme 16).

The reaction mediated by hypervalent iodine provides an alternative method for constructing glycosidic bonds of nucleoside derivatives by using a glycal as sugar donor. Its usefulness was proved by applying the reaction to synthesize new nucleoside derivatives as mentioned above.

### Synthesis of acyclic nucleosides

It is known that the oxidative C–C bond cleavage of glycols, epoxides, and olefins takes place by the action of hypervalent iodine [38,71–72]. For example, Havare and Plattner reported the oxidative cleavage of α-aryl aldehydes using iodosylbenzene to give chain-shortened carbonyl compounds and formaldehyde [71]. In the field of carbohydrate chemistry, similar deformylation by action of hypervalent iodine has also been demonstrated: the β-fragmentation reaction of an anomeric alkoxy radical of carbohydrates was mediated by a hypervalent iodine reagent [73]. The reaction results in the formation of carbohydrates with a reduction of one carbon. From the viewpoint of the synthetic method, the reaction would be useful for dehomologation of aldoses and preparation of chiral synthons deriving from sugars. The reaction procedure involves the initial formation of an alkoxy anomeric radical by a hypervalent iodine reagent in the presence of iodine, which triggers the β-fragmentation of the C1–C2 bond. As a result, a C2 radical is generated and is further oxidized to a carbocation that is reacted with nucleophilic agents to give the desired products.

**Scheme 17:**
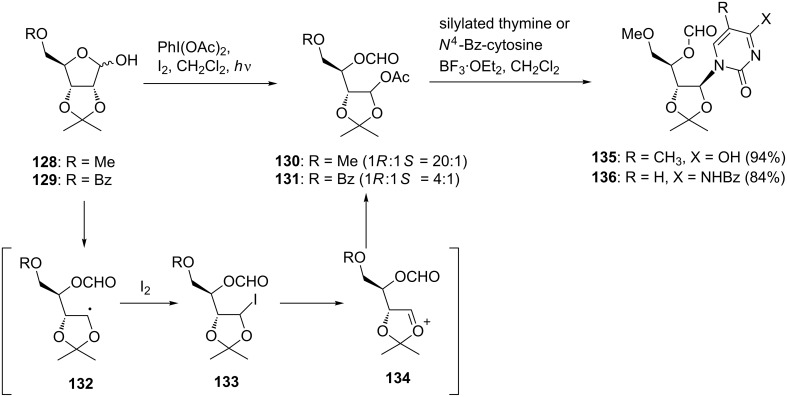
Synthesis of acetoxyacetals using hypervalent iodine and addition of silylated base.

Boto et al. applied the reaction to the one-pot synthesis of acyclic nucleosides that belong to an important class of nucleosides with antiviral activity [74]. First, they tried to synthesize acyclic nucleosides in a stepwise manner. The substrates **128** and **129** for the fragmentation reaction were synthesized from ribose in a few steps by the conventional method. The oxidative scission of **128** and **129** was carried out by treatment with diacetoxyiodobenzene and iodine under irradiation with visible light to give acetoxy acetals **130** and **131** in good yields with high stereoselectivities. As shown in Scheme 17, the reaction was expected to proceed via the formation of anomeric alkoxyl radicals, which underwent fragmentation to produce radical **132**. The radical **132** could be trapped with iodine, giving iodide **133**. The oxycarbenium ion **134** generated by the extrusion of iodide from **133** reacted with the acetoxy ion to furnish the resulting acetate derivatives. The acetates **130** and **131** were then treated with silylated thymine or *N*^4^-benzoylcytosine in the presence of a Lewis acid to give the desired acyclic nucleosides **135** and **136** in excellent yields. The results revealed that the nucleophilic attack of the nucleobase selectively occurred from the less hindered side of the oxycarbenium ion intermediates, giving 1’,2’-*trans* isomers as major products (Scheme 17).

**Scheme 18:**
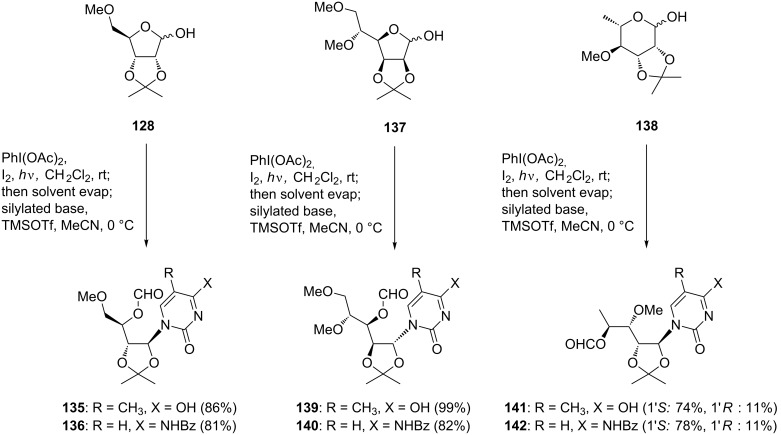
One-pot fragmentation-nucleophilic additions mediated by hypervalent iodine.

Based on the conditions for the stepwise fragmentation and glycosylation procedure, Boto et al. explored the one-pot version of the reaction [74]. When the β-fragmentation, the first step of oxidative glycosylation, was carried out in CH_2_Cl_2_ and then the Lewis acid and the silylated base were added, the acyclic nucleosides were obtained in low yields. Boto and co-workers overcame this problem by replacing the solvent before glycosylation. After the fragmentation reaction was finished, the solvent (CH_2_Cl_2_) was removed and replaced with acetonitrile. The resulting mixture was treated with TMSOTf and the silylated base. Under the optimized conditions, the reactions of ribose derivative **128**, mannose derivative **137**, and rhamnose derivative **138** gave the desired acyclic nucleosides in excellent yields as shown in Scheme 18. It is worth noting that the overall yields for the one-pot process are comparable or superior to those obtained with the two-step procedure (Scheme 18).

### Synthesis of disaccharides

Classically, carbohydrates have been considered primarily an energy source for life – as in the cases of glucose, fructose and their oligosaccharides, e.g., starch. However, more recently it has been revealed that oligosaccharides and glycoconjugates also play important roles in various biological processes, as mentioned earlier. As a result, the increasing significance of oligosaccharides in biological events has led to a strong demand for synthetic routes towards oligosaccharides, which would also contribute to the identification and development of drug candidates. For example, cancer immunotherapy based on vaccines derived from carbohydrate antigen–adjuvant combinations has received much attention in recent years [75–77]. However, the difficulties associated with the isolation of tumor-associated carbohydrate antigens from natural sources have impeded extensive research. Thus, the most promising approach to the supply of these antigens is to develop a suitable method for their chemical synthesis.

**Figure 7:**
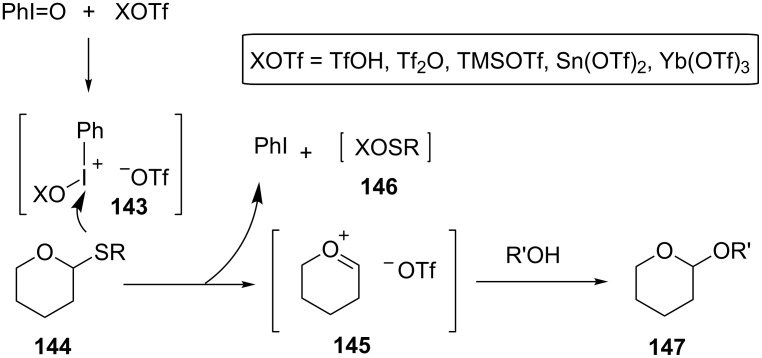
The reaction of thioglycoside with hypervalent iodine in the presence of Lewis acids.

To date, various glycosylation reactions capable of constructing oligosaccharides with high stereoselectivities have been reported [18–19]. Thioglycosides are often used as a sugar donor in these reactions due to their stability under various conditions and specific activation with thiophilic agents. For example, one of the typical conditions used for the construction of oligosaccharides is the combination of Lewis acids and iodine or its chemical equivalents. Fukase and co-workers reported a glycosylation reaction with thioglycoside using hypervalent iodine reagents in the 1990s [78–79]. The outline and postulated mechanism of the reaction are shown in Figure 7. The reaction of iodosylbenzene and electrophiles, e.g., triflic anhydride or Lewis acids, should generate a potent thiophile **143** that reacts with thioglycoside **144** to form an oxocarbenium ion **145**. The resulting oxocarbenium ion **145** should in turn react with a sugar acceptor to give the glycosylated product **147** (Figure 7).

**Scheme 19:**
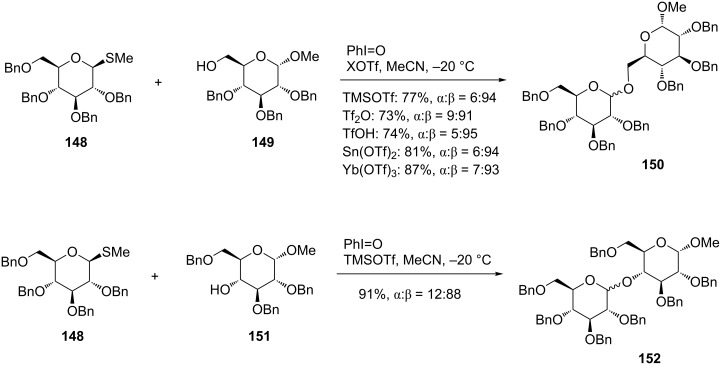
Synthesis of disaccharides employing thioglycosides under an oxidative coupling reaction mediated by hypervalent iodine.

By this reaction, Fukase et al. reported the glycosylation of methyl thioglycoside **148** as a sugar donor to give disaccharides **150** and **152** in high chemical yields as depicted in Scheme 19. As mentioned above, not only triflic anhydride, but various Lewis acids (TMSOTf, Sn(OTf)_2_, Yb(OTf)_3_) and a Brønsted acid (TfOH) were proven useful as activators, by which the reaction finished in a short time and gave the products with high stereoselectivity [79].

**Scheme 20:**
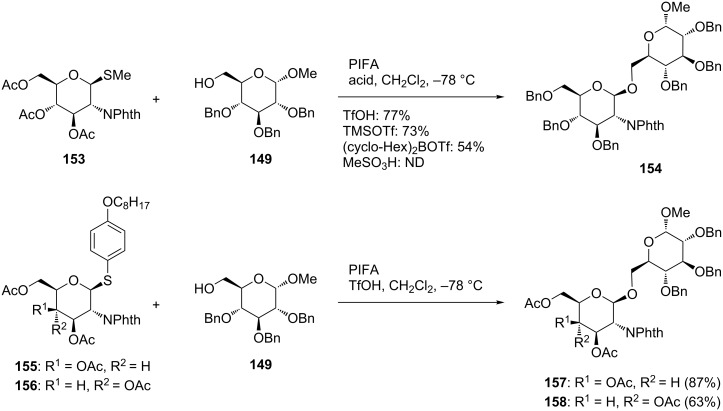
Synthesis of disaccharides using disarmed thioglycosides by hypervalent iodine-mediated glycosylation.

Recently, the reaction was revisited by Kajimoto et al., who sought a glycosylation reaction that could be applied to disarmed thioglycosides using hypervalent iodine reagents [80–81]. One of the reactions they examined was the glycosylation reaction of methyl 2-phthalimidothioglucopyranoside **153** with methyl tribenzylglucopyranoside **149** by PIFA in the presence of various acid catalysts. The results showed that the reaction with PIFA and TfOH afforded the best result, giving disaccharide **154** in 77% yield. On the other hand, the use of bis[cyclohexyl]trifluoromethanesulfonylborane [(cyclo-Hex)_2_BOTf] and methanesulfonic acid resulted in a poor yield. The synthesis of disaccharides under the optimized conditions was performed using “odorless” thioglycoside **155** and **149** as the donor and the acceptor [81]. Even with the combination of “disarmed” **155** and “armed” **149**, the reaction gave rise to the desired disaccharide **157** in 87% yield. The same reaction of the corresponding 3-epimer **156** proceeded smoothly to give the disaccharide **158** in good yield (Scheme 20).

**Scheme 21:**
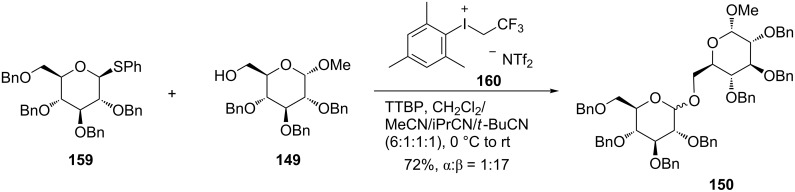
Glycosylation using aryl(trifluoroethyl)iodium triflimide.

Randolph and Danishefsky reported a glycal assembly strategy to the synthesis of a branched oligosaccharide [82]. Bennett and co-workers reported that phenyl(trifluoroethyl)iodonium triflimide was a stable promoter for glycosylation reactions using thioglycoside donors [83]. Since the reactions often were unselective in the absence of C2 acetate-directing groups, Bennett et al. investigated the compatibility of the above-mentioned reaction in nitrile solvents documented to have a β-directing effect, with the aim of developing a glycosylation that can be selectively achieved in the absence of directing groups. After preliminary screens, they found that the reaction in the presence of phenyl(trifluoroethyl)iodonium triflimide **160** and the non-nucleophilic base 2,4,6-tri-*tert*-butylpyrimidine (TTBP) at 0 °C with the solvent combination of 2:1 CH_2_Cl_2_/pivalonitrile provided the optimal reaction outcome. However, they also encountered a problem: the reduced solubility of substrate in the solvent system resulted in lower yields. They therefore examined mixed nitrile solvents again, and eventually found that a quaternary solvent mixture composed of 6:1:1:1 CH_2_Cl_2_/acetonitrile/isobutyronitrile/pivalonitrile greatly improved both the chemical yields and stereoselectivity, as shown in Scheme 21. The results suggested that both the solvent system and iodonium salt promoter are required for selectivity.

**Figure 8:**
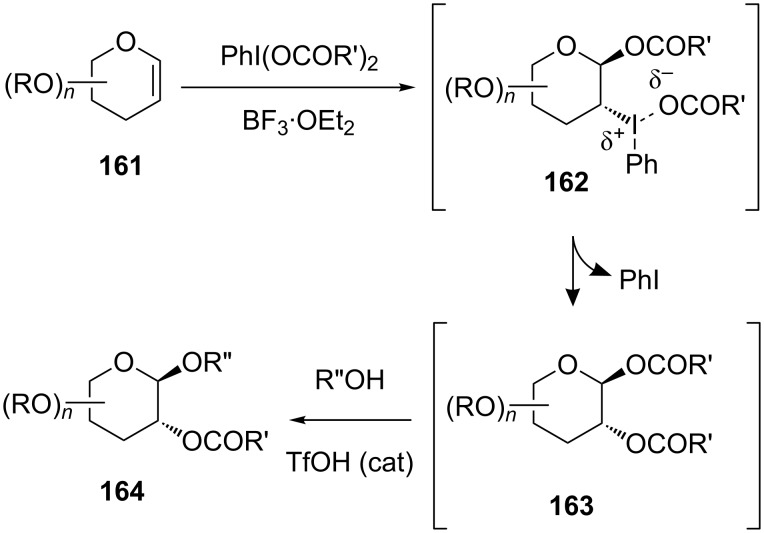
Expected mechanism of hypervalent iodine-mediated glycosylation with glycals.

Even though glycals have a π-electron-rich enol ether unit, reports regarding transformations involving glycal oxidation as well as installation of heteroatom substituents at the C2 position were limited. In 2001, Gin’s group reported the C2-acycloxyglycosylation procedure based on hypervalent iodine chemistry [84]. In this reaction, the use of a combination of hypervalent iodine and Lewis acid was key, as in the reactions described above. In this procedure, a solution of the glycal donor and a (diacyloxyiodo)benzene reagent was first treated with BF_3_·OEt_2_. Then, the glycosyl acceptor (R”OH) and a catalytic amount of TfOH were added to the mixture, giving the 1,2-*trans* disubstituted C2-acyloxylglycoside. A plausible mechanism of the reaction is shown in Figure 8. The first step of the reaction between glycal **161** and (diacyloxyiodo)benzene formed the glycosyl ester intermediate **162** bearing a phenyl iodonium(III) functionality at C2, which was transformed to a diacyloxylated product **163**. As evidence in support of this mechanism, they reported that **163** was indeed isolated when the reaction was finished at the first step. In the second step, the resulting diacyloxylated product **163** could effectively glycosylate the appropriate acceptor by the action of TfOH to give the C2-acyloxyglycoside **164** with good selectivity at the anomeric position as a consequence of participation by the neighboring C2 acyloxy group (Figure 8).

**Scheme 22:**
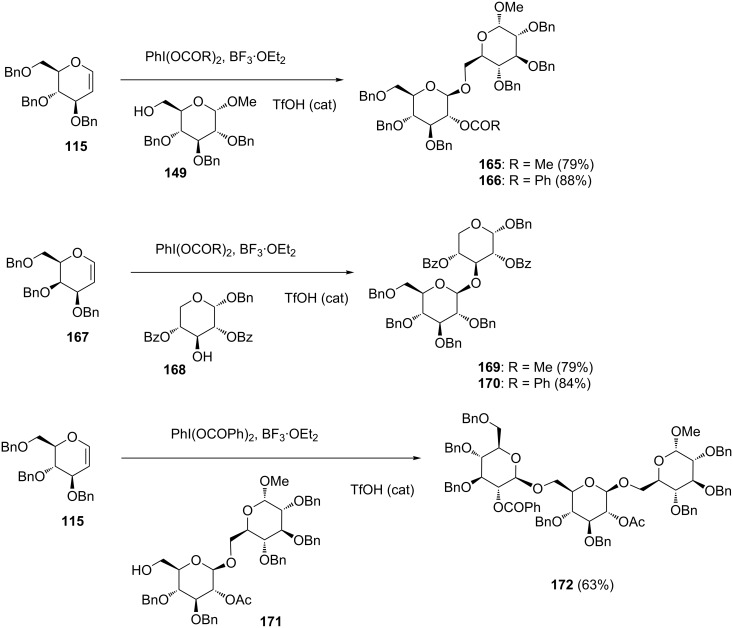
Synthesis of oligosaccharides by hypervalent iodine-mediated glycosylation with glycals.

They prepared C2-acyloxy glycosides **165**, **166**, **169**, **170** and **172** using hypervalent iodine-mediated coupling reactions with glycals, and the results are shown in Scheme 22. Either (diacetoxyiodo)benzene or (dibenzoyloxyiodo)benzene could serve as an efficient oxidant, and the reactions utilizing them gave the products installing either the acetate or benzoate functionality, respectively, at the C2-position. Both glucal **115** and galactal **167** were amenable to the oxidative glycosylation reaction to stereoselectively give C2-acyloxylated β-glycosides in good yields [84] (Scheme 22).

**Scheme 23:**
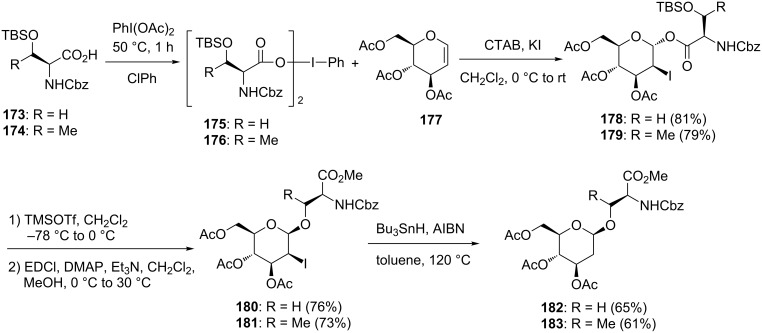
Synthesis of 2-deoxy amino acid glycosides.

Hotha and co-workers utilized the reaction of glycals with hypervalent iodine reagents for the stereoselective synthesis of C2 deoxyglycosides and amino acid glycoconjugates [85]. In their work, they also utilized an important chemical attribute of cetylammonium bromide (CTAB) – namely, CTAB forms surfactant-assembled lipophilic nanoreactors stable in organic solvents, which could be used for regioselective functionalization of indenes. Therefore, they investigated the regioselective iodination of glycals by using CTAB and hypervalent iodine reagents for the synthesis of 2-deoxy-2-iodoacetates. In the preliminary experiments, the reaction between per-*O*-acetylglucal (**177**) and PhI(OAc)_2_ in CTAB and KI gave *trans-*2-iodo α-acetate and its corresponding bromo acetate in a 94:5 ratio. The latter was expected to be formed by halide counter ion exchange between CTAB and KI. Since the reaction occurred as expected, it was applied to the synthesis of amino acid conjugates. Acetyl groups of the (diacetoxyiodo)benzene were exchanged with N- and O-protected amino acids by slow evaporation of a mixture of PhI(OAc)_2_ and amino acid **173** and **174** in chlorobenzene to give PhI(OCOR)_2_ compounds **175** and **176**. The formation of iodo ester glycosides **178** and **179** from **175** and **176** was achieved in very good yields under the conditions shown in Scheme 23.

Notably, the resulting iodo ester glycosides **178** and **179** were considered to have self-assembled structures versatile for the synthesis of serenylated and threonylated glycosides by intramolecular glycosylation. In addition, the access to 2-deoxyglycosides should be easily achievable by subsequent radical deiodination of the products. After several experiments, treatment with a catalytic amount of TMSOTf was found to be suitable for the intramolecular glycosylation, giving the corresponding acid, which was easily converted to the corresponding methyl ester **180** under EDCI/DMAP/MeOH conditions [85]. Similarly, the reaction of the threonine derivative **179** afforded **181** in good yield. Radical deiodination of **180** and **181** using Bu_3_SnH and AIBN successfully gave 2-deoxy-β-glycosides **182** and **183**, which were difficult to synthesize from the corresponding 2-deoxy sugar derivative in a stereoselective manner (Scheme 23).

**Figure 9:**
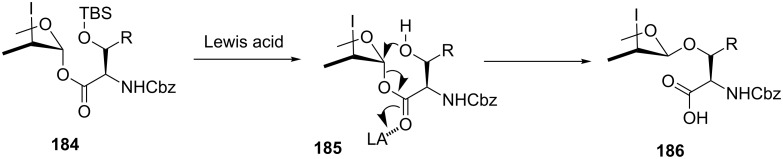
Rationale for the intramolecular migration of the amino acid unit.

As mentioned above, the iodo ester glycosides were considered to have self-assembled structures suitable for intramolecular glycosylation. As depicted in Figure 9, treatment of **184** with TMSOTf first cleaved the silyl ether to form **185**, which was correctly positioned to undergo intramolecular glycosidation. As a result, the Lewis acid could also facilitate the departure of the anomeric ester and the resulting **185** gave rise to the intramolecular nucleophilic attack to furnish the corresponding acid **186**.

Glycals and thioglycosides were often used as sugar donors for the glycosylation of oligosaccharides. It is interesting that the hypervalent iodine-mediated oxidative reactions with theses derivatives provide a different method to build glycosidic bonds. Diversity in glycoside bond forming reactions would contribute to improve the oligosaccharide synthesis.

## Conclusion

The Pummerer-type glycosylation includes oxidation of a sulfide to the corresponding sulfoxide followed by the TMSOTf-mediated coupling reaction. The reaction utilizing hypervalent iodine reagents could bypass one step of the Pummerer-type glycosylation and directly give 4’-thionucleosides from the corresponding sulfide derivative. The reaction could be efficiently applied to the synthesis of 4’-selenonucleosides as well as 4’-thionucleosides. Based on the concept of hypervalent iodine-mediated glycosylation, a reaction applicable to the synthesis of carbocyclic nucleosides and a coupling reaction between nucleobase and glycal derivatives were developed. The latter reaction was employed to synthesize dihydropyranonucleosides. Oxidative scission is a characteristic reaction mediated by hypervalent iodine reagents and is typically used for dehomologation of sugars. A one-pot glycosylation using this reaction was also developed for the synthesis of acyclic nucleoside derivatives. In addition to nucleoside synthesis, hypervalent iodine-mediated glycosylation could also be applied to the synthesis of oligosaccharides and glycoconjugates when thioglycosides and glycals were used as sugar donors. There is no doubt that the use of hypervalent iodine reagents greatly improved the efficiency of the synthesis of nucleosides and oligosaccharides. The results of these syntheses demonstrate the power of glycoside bond-forming reactions, and should assist in the future identification or synthesis of biologically active nucleoside and glycoconjugate derivatives.
